# Targeting Estrogen Receptor Beta Ameliorates Diminished Ovarian Reserve via Suppression of the FOXO3a/Autophagy Pathway

**DOI:** 10.14336/AD.2024.0221

**Published:** 2024-02-21

**Authors:** Fangyuan Li, Jingwen Zhu, Jinchen Liu, Yongyan Hu, Peili Wu, Cheng Zeng, Ruihui Lu, Ning Wu, Qing Xue

**Affiliations:** ^1^Department of Obstetrics and Gynecology, Peking University First Hospital, Beijing, China; ^2^Department of Obstetrics and Gynecology, Beijing Tsinghua Changgung Hospital, Beijing, China; ^3^Laboratory Animal Center, Peking University First Hospital, Beijing, China

**Keywords:** DOR, Estrogen receptor beta, PHTPP, Autophagy, FOXO3a

## Abstract

Diminished ovarian reserve (DOR) refers to a decrease in the number and/or quality of oocytes, leading to infertility, poor ovarian response and adverse pregnancy outcomes. Currently, the pathogenesis of DOR is largely unknown, and the efficacy of existing therapeutic methods is limited. Therefore, in-depth exploration of the mechanism underlying DOR is highly important for identifying molecular therapeutic targets for DOR. Our study showed that estrogen receptor beta (ERβ) mRNA and protein expression was upregulated in granulosa cells (GCs) from patients with DOR and in the ovaries of DOR model mice. Mechanistically, elevated ERβ promotes forkhead transcription factor family 3a (FOXO3a) expression, which contributes to autophagic activation in GCs. Activation of FOXO3a/autophagy signalling leads to decreased cell proliferation and increased cell apoptosis and ultimately leads to DOR. In a cyclophosphamide (Cy)-induced DOR mouse model, treatment with PHTPP, a selective ERβ antagonist, rescued fertility by restoring normal sex hormone secretion, estrus cycle duration, follicle development, oocyte quality and litter size. Taken together, these findings reveal a pathological mechanism of DOR based on ERβ overexpression and identify PHTPP as a potential therapeutic agent for DOR.

## INTRODUCTION

Diminished ovarian reserve (DOR) usually refers to a decrease in the quality and/or number of oocytes1. DOR can further develop into premature ovarian insufficiency (POI), which is a primary cause of female infertility2. The prevalence of DOR in infertile women is estimated to be between 6% and 64% in different age groups3. DOR is one of the most difficult challenges for assisted reproductive technology (ART) specialists due to the low live birth rate (LBR) and high cancellation rate 4. The etiology of DOR is thought to be related to genetic, autoimmune, or iatrogenic factors5. However, the etiology of DOR has yet to be fully elucidated. Thus, it is important to investigate the pathogenesis of DOR to identify therapeutic molecular targets for DOR and POI.

DOR is a reproductive system disease caused by a decrease in the quality and quantity of oocytes. Gap junctions are present between granulosa cells (GCs) and oocytes, which facilitate communication between them. The estrogen receptors are mainly expressed on GCs and not on oocytes. Therefore, GCs rely on these receptors to regulate oocyte development and maturation more effectively 6. Accumulating evidence has confirmed that follicular atresia is triggered by GC apoptosis7-9, and a previous study reported that the GC apoptosis rate was significantly increased in patients with DOR10. In addition to apoptosis, autophagy has been shown to be involved in follicular loss11. Autophagy is a self-degradation process responsible for the disassembly of damaged cell components and recycling of bioenergetic molecules and depends on lysosomes12. The formation of autophagosomes involves a variety of proteins and complexes and is also regulated by a variety of genes. Autophagy-related genes (ATGs) and Beclin1 are important regulatory genes involved in autophagosome maturation. LC3 is a key structural protein of autophagosomes, and the expression of ATG7, BECLIN1 and LC3 can reflect autophagic activity to a certain extent13. Increased autophagic signalling during follicular atresia was observed in rat GCs14. Recent studies have shown that autophagy is involved in regulating GC apoptosis and accelerating follicular atresia15, 16. This evidence highlights the importance of GC death in the pathogenesis of DOR. However, the underlying mechanism of GC death in DOR remains to be determined.

FOXO3a is a member of the forkhead transcription factor (FOXO) family and plays an important role in the cell cycle, apoptosis, DNA damage repair, oxidative stress, and autophagy17. Studies have reported that FOXO3a can activate autophagy by regulating the expression of autophagy-regulating genes18, 19. FOXO3a can detect and correct autophagic flux dysfunction, ensuring that cells lacking autophagic capacity undergo apoptotic death20. Furthermore, many studies have shown that FOXO3a is also associated with follicular development. Some researchers have suggested that the FOXO3 protein regulates follicle growth and atresia by promoting apoptosis in mammalian ovarian GCs and oocytes13, 21, 22. However, the relationship between FOXO3a and autophagy in ovarian reserve function needs to be further explored.

Estrogen plays an important role in follicular development23. There are two subtypes of estrogen receptor (ER), estrogen receptor alpha and beta (ERα and ERβ), which are encoded by the estrogen receptor 1 (ESR1) and 2 (ESR2) genes, respectively. Among them, ERβ is the main ER subtype in the ovary24 and is abundantly expressed in GCs25, but there is no consensus on the role of ERβ in GCs or follicle development. The autophagy-inducing function of ERβ has been demonstrated in several cell types, such as breast cancer cells, human seminoma cells, and osteosarcoma cells26-29. Moreover, in prostate cancer, ERβ causes apoptosis by transactivating FOXO3a30. Currently, there are no consistent findings related to the effects of ERβ on oocytes and GCs in follicles, and additional research is needed to confirm whether ERβ affects female fertility. However, the relationships between ERβ and FOXO3a and between ERβ and autophagy in GCs have not been determined, and the effects of ERβ on ovarian reserve and female fertility still need to be explored.

Here, we found that overexpression of ERβ activated apoptosis through the FOXO3a/autophagy signalling pathway in GCs and ultimately led to DOR. Subsequently, in a DOR mouse model, the ERβ antagonist PHTPP was shown to reverse the pathogenesis of DOR, restore the number and quality of oocytes, promote embryonic development, and increase the LBR. The above study provides an experimental basis for ERβ as a possible molecular marker and a therapeutic target for DOR.

## MATERIALS AND METHODS

### Clinical samples

The study was approved by the Institutional Review Board of Peking University (No. 2020 [184]), and informed consent was signed by all participants. Twenty-three women with DOR who received in vitro fertilization or intracytoplasmic sperm injection and embryo transfer (IVF/ICSI-ET) at Peking University First Hospital were included. The diagnostic criteria for DOR were (i) age <40 years, (ii) anti-Müllerian hormone (AMH) level <1.1 ng/mL, and (iii) an ovarian antral follicle count (AFC) <6. Women with a history of ovarian surgery, radiotherapy, chemotherapy, or known chromosomal abnormalities were excluded. Twenty-three women seeking infertility treatment due to male factors or tubal obstruction were enrolled as controls. The inclusion criteria were as follows: i) age <40 years, (ii) 1.4 ng/mL<AMH<4 ng/mL, (iii) 6≤AFC<15, and (iv) basal serum follicle-stimulating hormone (FSH) ≥10 IU/l. The inclusion criteria were based on Chinese expert consensus on the clinical diagnosis and treatment of early-onset ovarian insufficiency in 2017 and the Bologna standard. The clinical characteristics of the participants are summarized in Table 1.

### GC isolation

Follicular aspirates obtained from each participant during oocyte retrieval were centrifuged at 3000 rpm at 4°C for 10 minutes, after which the supernatant was removed. Then, phosphate-buffered saline (PBS) was added, and the samples were gently mixed and centrifuged at 4°C and 3000 rpm for 10 min. The precipitates containing human ovarian GCs were washed with PBS once and then resuspended in 1 mL of PBS. Afterwards, 2 mL of Percoll solution was added to the mixture, and the mixture was centrifuged at 4°C and 2000 rpm for 30 min. Next, the second layer of liquid with GCs was transferred to a new centrifuge tube. After gentle mixing with PBS, the mixture was centrifuged at 3000 rpm for 10 min to collect the GCs. Finally, the isolated GCs were snap frozen and stored at -80°C until use.

**Table 1:** Baseline characteristics in the DOR and control groups.

Variable	Control	DOR	t/χ2	P
**Embryo transfer cycles (n)**	23	23		
**Age (years)**	33.3±3.0	35.0±3.3	-1.883	0.339
**Duration of infertility (years)**	3.5±2.4	3.1±2.0	0.696	0.633
**BMI (kg/m2)**	21.4±2.4	21.7±3.7	0.681	0.222
**Primary infertility rate (%)**	17(73.9)	16(69.6)	0.107	0.743
**AMH (ng/mL)**	2.6±0.9	0.5±0.3	11.248	<0.001
**Basal FSH (U/L)**	7.4±1.6	13.2±9.9	-2.783	0.001
**Basal LH (U/L)**	4.0±1.6	6.5±5.9	-1.971	0.011
**Basal E2 (pmol/L)**	42.3±21.9	54.8±59.7	-0.943	0.112
**Basal FSH/LH**	2.5±1.5	2.7±1.9	-0.373	0.922
**AFC (n, bilateral)**	11.1±3.4	4.0±1.9	8.873	0.002
**Number of oocytes retrieved(n)**	10.2±5.0	2.5±2.1	6.788	0.001

BMI, body mass index; AMH, anti-Müllerian hormone; AFC, antral follicular count; E2, oestradiol; FSH, follicle-stimulating hormone

### Cell culture

The human ovarian GC line KGN was generously donated by Dr. Jie Qiao of Peking University Third Hospital, Beijing, China, in 2019. KGN cells were cultured in DMEM/F12 supplemented with 1% penicillin-streptomycin and 10% foetal bovine serum in a humidified atmosphere of 5% CO2 at 37°C.

### Transient cell transfection

On the day before transfection, KGN cells were seeded in 6-well plates. When the cell density reached 70% confluence, only DMEM/F12 was used to replace the medium, and a mixture of transfection reagent containing Opti-MEM, ERβ siRNA, FOXO3a siRNA or negative control siRNA (GenePharma, Shanghai, China) and Lipofectamine RNAiMAX (Invitrogen) were added dropwise to each well. After 6 h, the culture medium was replaced with normal culture medium, and after an additional 48 h, the cells were collected after culture. The siRNA sequences used were as follows: ERβ sense: ATGATCAGCTGGGCCAAGAA (5′ to 3′); ERβ antisense: CCACATCAGCCCCATCATTAA (5′ to 3′); FOXO3a sense: CGGACAAACGGCTCACTCT (5′ to 3′); and FOXO3a antisense: GGACCCGCATGAATCG ACTAT (5′ to 3′). For overexpression of ERβ and FOXO3a, the empty pENTER plasmid, empty pCMV plasmid, pCMV-ESR2 plasmid, or pENTER-FOXO3a plasmid (WZ Biosciences, Shangdong, China) was transfected into KGN cells with Lipofectamine 3000 transfection reagent (Invitrogen, Carlsbad, CA) according to the manufacturer’s protocol. The procedure was similar to that described above. Cells were harvested for RNA or protein isolation after 48 h of incubation.

### Quantitative real-time PCR (qPCR)

Total RNA was extracted using TRIzol reagent (Invitrogen) according to the manufacturer’s instructions. The RNA was reverse transcribed into cDNA using an ABI High-Capacity cDNA Archive Kit (Applied Biosystems, Foster City, California), and real-time PCR was carried out on a 7500 Real-Time PCR System using 2× SYBR Green SuperMix (Invitrogen). The expression levels of the target genes relative to the internal parameters were calculated by the 2-△△Ct method as follows: △Ct = △Cttarget - △Ctreference, - △△Ct = - (△Cttreat - △Ctcontrol). The specific primers used for qPCR analysis are shown in Table 2. The primer specificity was verified by melting curve analysis. Actin was used as an internal reference gene.

### Western blotting

The cells were lysed in RIPA buffer (Solarbio, Beijing, China) supplemented with 1 mM PMSF (Solarbio), and a BCA protein concentration assay kit (Solarbio) was used to measure the protein concentrations. Equal amounts of protein were separated via SDS-PAGE on a 10% gel and electroblotted onto polyvinylidene fluoride membranes (Millipore, Billerica, MA, USA). The membranes were blocked with 5% nonfat dry milk (Sangon Biotech, Shanghai, China) for 1 h and probed overnight at 4°C with primary antibodies. Anti-ERβ (04842, 1:1000) was obtained from Merck Millipore. Anti-FOXO3a (D19A7, 12829, 1:1000), anti-Beclin1 (D40C5, 3495T, 1:1000) and anti-ATG7 (D12B11, 8558, 1:1000) were purchased from Cell Signaling Technology (MA, USA). Anti-LC3 (ab48394, 1:1000) and anti-cyclin D1 (ab16663, 1:1000) were purchased from Abcam (Cambridge, UK). Anti-BCL2 (A19693, 1:1000) and anti-BAX (A19684, 1:1000) were obtained from ABclonal (Wuhan, China). Anti-GAPDH (TA-08, 1:1000) and anti-actin (TA-09, 1:1000) were purchased from ZSGB-BIO (Beijing, China). After five washes in TBST, the membranes were incubated with a secondary antibody, anti-mouse/rabbit IgG peroxidase-conjugated secondary antibody (Santa Cruz, CA, USA), conjugated with horseradish peroxidase for 1 h at 37°C. The protein bands were visualized with enhanced chemiluminescence (ECL) solution (Syngene, Cambridge, United Kingdom). ImageJ software was used to analyse the intensities of the Western blot bands. The results were normalized with housekeeping genes.

**Table 2:** qPCR primers.

Gene	Direction	Primer
ERβ	Forward	5'-ATGATCAGCTGGGCCAAGAA-3'
	Reverse	5'-CCACATCAGCCCCATCATTAA-3'
FOXO3a	Forward	5'-CGGACAAACGGCTCACTCT -3'
	Reverse	5'-GGACCCGCATGAATCGACTAT-3'
Beclin1	Forward	5'-CCATGCAGGTGAGCTTCGT-3'
	Reverse	5'-GAATCTGCGAGAGACACCATC-3'
ATG7	Forward	5'-CTGCCAGCTCGCTTAACATTG-3'
	Reverse	5'-CTTGTTGAGGAGTACAGGGTTTT-3'
CyclinD1	Forward	5'-CTGTGCTGCGAAGTGGAAACCAT-3'
	Reverse	5'-TTCATGGCCAGCGGGAAGACCTC-3'
BCL2	Forward	5'-AAACTTGACAGAGGATCATGCTGTA-3'
	Reverse	5'-TGGCATGAGATGCAGGAAATT-3'
BAX	Forward	5'-CCCGAGAGGTCTTTTTCCGAG-3'
	Reverse	5'-CCAGCCCATGATGGTTCTGAT-3'
Actin	Forward	5'-GTGGCCGAGGACTTTGATTG-3'
	Reverse	5'-CCTGTAACAACGCATCTCATATT-3'
ERβ mouse	Forward	5'-TTCTTTCTCATGTCAGGCACA-3′
	Reverse	5'-CTCGAAGCGTGTGAGCATT-3′
FOXO3a mouse	Forward	5'-ACAAACGGCTCACTTTGTCCCAGA-3'
	Reverse	5'-TCTTGCCCGTGCCTTCATTCT-3'
Beclin1 mouse	Forward	5'-TGAAATCAATGCTGCCTGGG-3'
	Reverse	5'-CCAGAACAGTATAACGGCAACTCC-3'
ATG7 mouse	Forward	5'- TCTGGGAAGCCATAAAGTCAGG-3'
	Reverse	5'-GCGAAGGTCAGGAGAA-3'
Cyclin D1 mouse	Forward	5'-TCAAGTGTGACCCGGACTGC-3'
	Reverse	5'-CCTTGGGGTCGACGTTCTG-3'
BCL2 mouse	Forward	5'-CTTTGAGTTCGGTGGGGTC-3'
	Reverse	5'-GTTCCACAAAGGCATCCCA-3'
Actin mouse	Forward	5′-GTGACGTTGACATCCGTA-3′
	Reverse	5′-GTAACAGTCCGCCTA-3'

### Transmission electron microscopy

GCs and KGN were fixed with glutaraldehyde (G5882; Sigma-Aldrich) and osmium tetroxide and postfixed with OsO4 and sucrose. Then, graded concentrations of ethanol were used to dehydrate the cells. Subsequently, the samples were embedded in embed-812 dodecenylsuccinic anhydride, dimethylaminomethyl phenol and methylnadic anhydride. Then, the 80-nm-thick sections were contrasted with lead citrate and uranyl acetate. A JEM-1400plus electron microscope was used to capture images. According to previously reported guidelines31, autophagosomes, also referred to as initial autophagic vacuoles, typically have a double membrane or a membrane at least partly visible as two parallel membrane bilayers under an electron microscope. Three patients were randomly selected from the DOR and control groups, and autophagosomes in 15 different cells per patient were observed and counted by Lei Zhang, an experienced professor from Peking University. A total of 3 independent experiments were performed, and autophagosomes in 15 different KGN cells were observed and counted in each experiment.

### Chromatin immunoprecipitation (ChIP)

KGN cells were cultured in 15-cm dishes. When the cell confluence reached 90%, a ChIP assay kit (Pierce Chromatin Prep Module; Thermo Scientific, MA, USA) was used according to the manufacturer's instructions. Briefly, cells were collected in PBS containing 1% protease inhibitors and crosslinked with 1% formaldehyde. The crosslinked cells were then cleaved by micrococcal nuclease and digested by enzymes to cut genomic DNA. A total of 5 μL of the digested chromatin (10% input) was stored at -20 °C as the input control and was used for IP elution and DNA recovery. The remaining digested chromatin was immunoprecipitated with an anti-ERβ antibody (04842, 1:200, Merck Millipore) with rotation at 4 °C overnight with rotation, IP elution and DNA recovery. IgG was used as a negative control. The purified DNA was analysed by RT-qPCR. The sequences of the FOXO3a promoter primers used were as follows: (**F**) 5'-GCGTGCCTTGACGAG-3'; (**R**) 5'-CAAGCTAG TTCGACGCGTACAA-3'.

### Luciferase assay

KGN cells were transfected with expression vectors encoding pcDNA3.1-ERβ, the pGL3-FOXO3a promoter and the pGL3-FOXO3a site mutation promoter (Genomeditech, Shanghai, China) using Lipofectamine™ 3000 transfection reagent. A 2 μg firefly luciferase reporter structure containing a FOXO3a promoter site mutation and 30 ng of the Renilla luciferase reporter plasmid pRL-TK were used as internal controls for each transfection. The cells were harvested 48 h post-transfection, and luciferase activity was measured with a luciferase assay system (Promega, WI, USA). The firefly luciferase activity in each well was normalized to the Renilla luciferase activity. The measurements were performed on cultured cells from 3 different subjects and repeated twice.

### Cell treatment

After transfection with the indicated siRNA or plasmid for 24 h, KGN cells were treated with the autophagy inhibitor 3-methyladenine (3-MA) (5 mM, S2767, Selleck) or the autophagy inducer rapamycin (rapa) (100 nM, S1039, Selleck) for another 24 h, after which the following experiments were performed.

### Flow cytometry analysis

Apoptosis was assessed using an Annexin V-FITC Apoptosis Detection Kit (BD Biosciences, USA). The cells were harvested after treatment, washed twice with cold PBS and binding buffer, and incubated in the dark for at least 15 min with Annexin V-FITC. Propidium iodide (PI) was added to the cells, which were incubated in the dark at room temperature for 10 min. Apoptosis was determined via flow cytometry. The degree of early apoptosis was determined by flow cytometry as the percentage of cells that were positive for Annexin V and negative for PI. Cells negative for Annexin V and PI were classified as normal. Cells that were positive for Annexin V and negative for PI were considered early apoptotic cells. Annexin V- and PI-positive cells were considered late apoptotic cells.

### Cell Counting Kit-8 (CCK-8) assay

Cell viability was analysed by the CCK-8 assay (Beyotime, Shanghai, China) according to the manufacturer’s protocol. The cells were cultured at a density of 0.5×10^4^ cells/well in 100 μL of medium in 96-well microplates (Corning, USA). After 24 h, 10 μL of CCK-8 reagent was added to each well, and the cells were then cultured for 2 h. All the experiments were performed in triplicate. The absorbance was analysed at 450 nm using a microplate reader (Bio-Rad, Hercules, CA, USA), and wells without cells were used as blanks. The proliferation of cells was assessed by measuring the absorbance.

### Animal model

Female (7-8 weeks old) and male (8-9 weeks old) C57BL/6J mice and female (28-32 g) and male (10 weeks old) ICR mice were purchased from Beijing Vital River Laboratory Animal Technology Co., Ltd., and housed under speciffic pathogen-free (SPF) conditions at a controlled temperature (22 ± 1°C) and humidity (60 ± 10%) with ad libitum access to water and regular rodent chow on a 12 h light/12 h dark cycle. All animal protocols were approved by the Peking University First Hospital Animal Care and Use Committee (protocol number: J202141).

For construction of a cyclophosphamide (Cy)-induced DOR (Cy-DOR) model, 7- to 8-week-old C57BL/6 female mice were weighed and then given a single intraperitoneal injection of Cy (75 mg/kg, 200-300 μL) or an equal volume of saline as a control. Seven days later, we randomly divided the mice according to the conditions where the body weights of the mice in each group were matched. Then, the mice were administered PHTPP by intraperitoneal injection every other day for 14 days. The three groups were the DOR + PBS (vehicle), DOR + PHTPP (2 mg/kg, PHTPP2), and DOR + PHTPP (4 mg/kg, PHTPP4) groups. Serum and ovarian samples were collected after the mice were sacrificed. The weights of the mice and wet weights of the ovaries were measured (ovarian index = ovarian mass/mouse body weight × 100%).

### Evaluation of estrous cyclicity

Vaginal smears were collected from all of the mice daily at 9:00 am for 18 days and analysed via haematoxylin and eosin (H&E) staining. The stage of cyclicity was determined by the predominant cell type using microscopic analysis. The four phases of the oestrus cycle are predominantly characterized by different cell types: nucleated epithelial cells are the predominant cells in the proestrus phase; cornified epithelial cells are the predominant cells in the oestrus phase; equal numbers of leukocytes, cornified epithelial cells and nucleated epithelial cells are present in the metestrus phase; and leukocytes are the predominant cells in the diestrus phase.

Analysis of oestradiol (E2), FSH, luteinizing hormone (LH), and AMH levels by enzyme-linked immunosorbent assays (ELISAs)

After incubating for 2 h at room temperature, the mouse blood was centrifuged for 20 min at 2000 × g, after which the supernatant was collected. AMH, E2, FSH and LH levels were measured using an ELISA kit (Cusabio, Wuhan, China) according to the manufacturer’s protocol. The results were analysed using a microplate reader (Bio-Rad, Hercules, CA, USA).

### Histopathology and follicle counting

Ovaries were collected and fixed in 4% paraformaldehyde for 24 h. Then, the tissues were embedded in paraffin and sliced into 5 μm thick pieces for H&E staining. Follicles were counted in every ffifth serial section from ten mice in each group. The mean number per section was calculated. The follicles were counted at different stages while the sections were viewed under a Nikon digital microscope (Nikon, Melville, NY, USA). The follicles were classified as follows32: primordial follicles consisted of one layer of flattened GCs surrounding the oocyte; primary follicles consisted of one to two complete layers of cuboidal GCs; secondary follicles consisted of more than one layer of cuboidal GCs with no visible antrum around an oocyte; and antral follicles consisted of an oocyte and multiple layers of cuboidal GCs and contained one or more antral spaces, possibly with cumulus oophores and a thecal layer.

### Terminal deoxynucleotidyl transferase-mediated dUTP nick end labelling (TUNEL) staining

For analysis of apoptosis in ovarian tissue, deparaffinized tissue sections were permeabilized with 10 μg/mL proteinase K in 10 mM Tris HCl and subjected to TUNEL staining according to the manufacturer’s instructions (Proteintech, Wuhan, China). The samples were counterstained with 4′,6-diamidino-2-phenylindole (DAPI, Molecular Probes). Sections were observed, and images were captured with an epifluorescence microscope (Axio Imager 2, Carl Zeiss) by the imaging program ZEN.

### Superovulation induction and oocyte collection

For collection of MII oocytes, superovulation was induced in mice from the NC, Cy, and Cy+PHTPP groups by intraperitoneal injection of 10 IU of pregnant mare serum gonadotrophin (PMSG), followed by 10 IU of human chorionic gonadotropin (hCG) 48 h later. Cumulus-oocyte complexes (COCs) were collected from oviductal ampullae 15-17 h after hCG injection. MII eggs and cumulus cell complexes were collected from oviducts. After hyaluronidase (500 IU/mL) treatment, the cumulus mass was dissociated, and MII eggs were collected in M2 medium.

### IVF and embryo culture

Epididymal spermatozoa were retrieved from the cauda epididymis of 12-15-week-old C57BL/6J male mice. The sperm suspensions were incubated in 10% serum protein substitute (SPS)/human tubal fluid (HTF) at 37°C in 5% CO2 and 95% humidity for 90 min. Ovulated oocytes with intact cumulus masses were recovered from the oviducts and maintained in HTF. Then, capacitated sperm at a concentration of 1-2×106/mL were added to COC droplets and cultured at 37°C in 5% CO2 at 95% humidity. Twenty-four to twenty-eight hours later, the oocytes were removed and examined for fertilization. Fertilization was assessed by two pronuclei (2PN). Fertilized embryos were cultured to the blastocyst stage in M16 medium for 86-88 h, and development was evaluated every 24 h.

### Surgical transfer of embryos into recipient mothers

Female ICR mice mated with vasectomized male ICR mice were used as recipient mothers. The day after a vaginal plug was observed was considered Day 1 of pseudopregnancy. Mice were anaesthetized using inhaled isoflurane (Steve Laboratories, UK). The uterine horn was exposed via a dorsal incision using tweezers. With a glass micropipette, 12 embryos per horn were transferred into the oviduct in a small volume of culture medium (M2). The expected perinatal period after 2-cell embryo transfer was 19 days. Female surrogate ICR mice gave birth near the end of the expected perinatal period, and the litter size of each mouse was recorded for comparison between groups.

### Fertility assessment

C57BL/6J female mice were housed with demonstrably fertile C57BL/6 males (3 months old) at a 1:1 ratio, and the delivered offspring were assessed. The number of pups per litter and pup health were recorded and statistically analysed.

### Reactive oxygen species (ROS) level analysis

Intracellular ROS levels were analysed using a Reactive Oxygen Species Assay Kit (Thermo, USA) according to the manufacturer’s instructions. Briefly, a 5 μM MitoSOX working solution was prepared. Degranulated MII oocytes from different groups were placed in MitoSOX working solution and incubated in a 5% CO2 incubator at 37°C for 20-30 minutes in the dark. After being washed three times in M2 medium, the oocytes were observed under a Leica microscope. The fluorescence intensity (excitation at 488 nm) was measured. All images were taken using fixed microscope parameters, and the fluorescence intensity of each oocyte was analysed via ImageJ software.

### Meiotic spindle staining

Degranulated MII oocytes were fixed in 4% paraformaldehyde at room temperature for 30 min, permeabilized in 0.5% Triton X-100 for 20 min, blocked with 1% BSA dissolved in PBS and then incubated with an anti-α-tubulin antibody in a dark humidified chamber overnight at 4°C. The next day, the oocytes were washed with new BSA 3 times and incubated with an Alexa Fluor 488-conjugated secondary antibody (1:200, CST) for 1 h at room temperature (RT). Chromatin staining was performed by incubating the sections with 5 μg/mL Hoechst 33342 (Sigma-Aldrich) for 5 min at room temperature. To confirm the specificity of the staining, isotope controls, the normal mouse IgG (SC-2025, Santa Cruz) and normal rabbit IgG (AB-105-C, R&D), instead of antibodies to specific proteins, were included in each experiment. There was no clear staining from these isotope controls was detected, suggesting that the signals are specific. Spindle and chromosome morphology were observed under an inverted laser confocal microscope with a water or oil lens.

**Figure 1.**
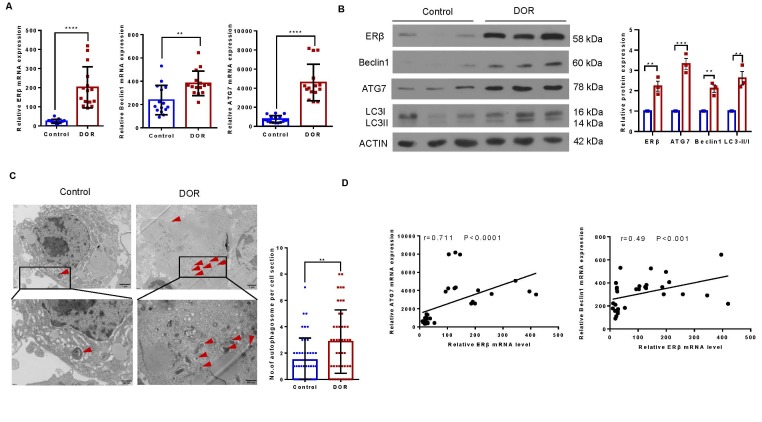
**GCs from patients with DOR exhibit aberrant autophagy and increased ERβ expression**. (**A**) ERβ, Beclin1 and ATG7 mRNA levels in GCs from the controls and patients with DOR were measured by qPCR (n = 15). (**B**) The expression of the ERβ, ATG7, Beclin1 and LC3 proteins in GCs from the controls and patients with DOR (n = 5) was assessed by Western blotting, and representative results are shown. ACTIN was used as the loading control. (**C**) Transmission electron micrographs showing characteristic autophagosome formation (red arrows) in GCs from the controls and patients with DOR. The number of autophagosomes was counted in 15 randomly selected cells per patient (n=3). (**D**) The correlation of ATG7 and Beclin1 expression with ERβ expression in GCs was analysed, and the linear regression coefficient and P value are shown (n=30). The data are presented as the mean ± SEM. Statistical analyses for mRNA expression and autophagosome counts were carried out by two-tailed Student's t test. The P value for protein expression between two groups was from Mann-Whitney U test. The linear regression coefficient and P value were carried out by Spearman correlation analysis (**P < 0.01; ****P < 0.0001).

### Statistical analysis

All the data are presented as the mean ± SEM. All the statistical analyses were conducted using SPSS 26.0 software. Kolmogorov-Smirnov test and Q-Q plot were used to test the normality of the data using. If N is too small to determine normality, we used the non-parametric alternative (Man-whitney U test for 2 samples or Kruskal-Wallis H test for more samples). Comparisons between two groups were performed using a two-tailed Student's t test. Comparisons among more than two groups were performed using one-way analysis of variance (ANOVA). Spearman correlation coefficients were calculated. P values < 0.05 were considered to indicate statistical significance.

**Figure 2.**
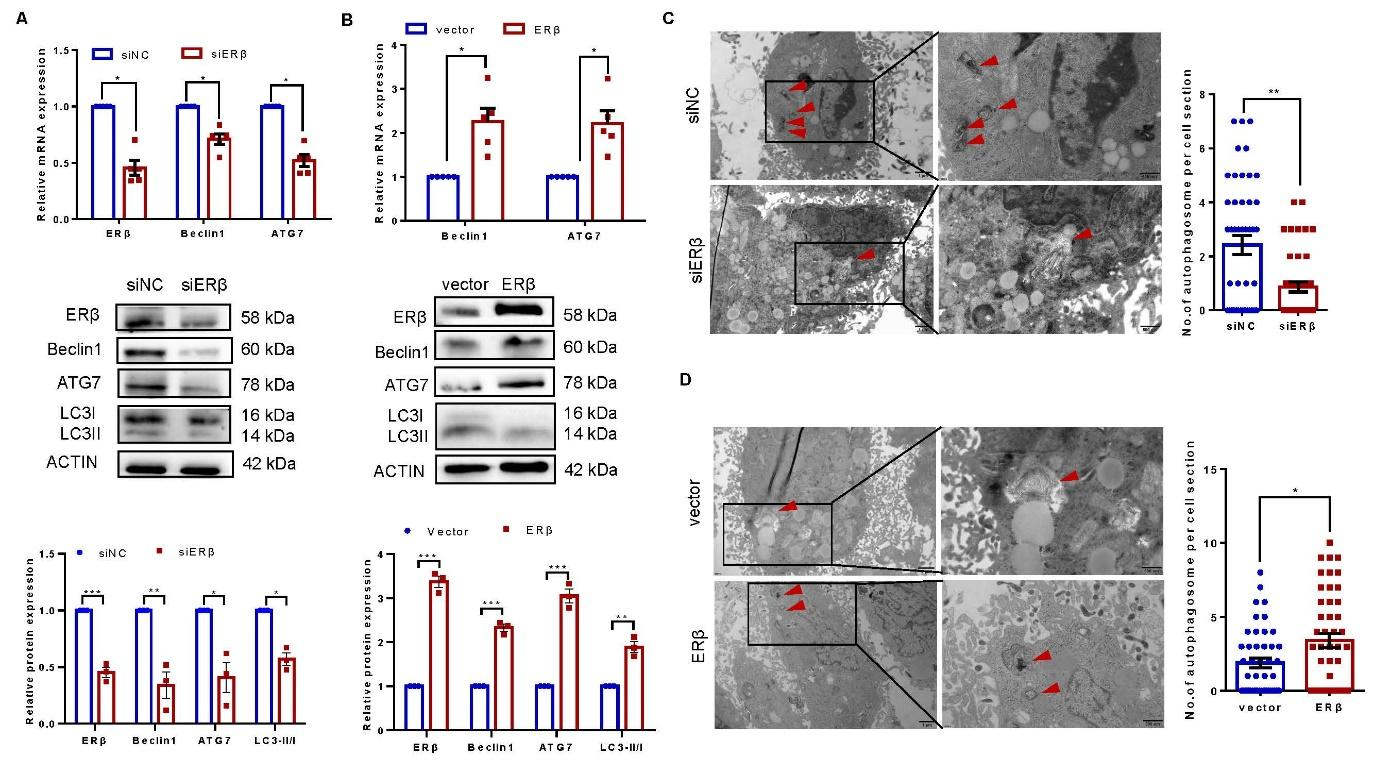
**ERβ induces autophagy in KGN cells**. (**A**) KGN cells were transfected with ERβ siRNA or control siRNA, and the levels of the indicated autophagy-related markers were measured via qPCR (n=5) or Western blotting (n=3). (**B**) KGN cells were transfected with an ERβ overexpression plasmid or vector, and the levels of the indicated autophagy-related markers were measured via qPCR (n=5) or Western blotting (n=3). The efficiency of the knockdown or overexpression was verified by Western blotting (n=3) or qPCR (n=5). (**C**) KGN cells were transfected with ERβ siRNA or control siRNA, and morphological examination was performed via transmission electron microscopy. The number of autophagosomes was counted in 15 randomly selected cells from each group (n=3). (**D**) KGN cells were transfected with the ERβ overexpression plasmid or vector, and morphological examination was performed via transmission electron microscopy. The number of autophagosomes was counted in 15 randomly selected cells in each group (n=3). The data are presented as the mean ± SEM. Statistical analyses were carried out by Mann-Whitney U test or two-tailed Student’s t test (*P < 0.05; **P < 0.01; ***P < 0.001).

## RESULTS

### GCs from patients with DOR exhibit increased expression of ERβ and autophagy

We examined the levels of ERβ and autophagy-associated proteins in DORs and control GCs to determine their relationship and found that ERβ expression was upregulated in GCs from patients with DOR. Moreover, upregulation of Beclin 1 (autophagy-related protein) and ATG7 (autophagy-related 7) and accumulation of the cleaved and lipidated form of MAP1LC3B-II/LC3B-II (microtubule-associated protein 1 light chain 3b) were also observed, indicating aberrant autophagic flux (Fig. 1A and B). Analysis via transmission electron microscopy analysis revealed that GCs from patients with DOR contained significantly more autophagosomes than did those from control patients (Fig. 1C). Moreover, a positive correlation was observed between ERβ expression and Beclin1 and ATG7 expression in GCs (Fig. 1D). These data indicate that elevated ERβ expression is positively correlated with the development of DOR associated with activated autophagy.

### ERβ induces autophagy in KGN cells

KGN cells were subjected to ERβ gain-of-function and loss-of-function assays to examine the effects of ERβ on autophagy-related gene expression and autophagosome formation in GCs. We observed a decrease in the expression of autophagy-related genes, including ATG7, Beclin1, and LC3, at both the mRNA and protein levels upon ERβ knockdown, while ERβ overexpression induced their expression (Fig. 2A and B). Transmission electron microscopy revealed that ERβ overexpression increased the number of autophagosomes in KGN cells, while fewer autophagosomes were found in ERβ knockdown cells (Fig. 2C and D). Taken together, these findings suggest that ERβ enhances autophagy in KGN cells.

**Figure 3.**
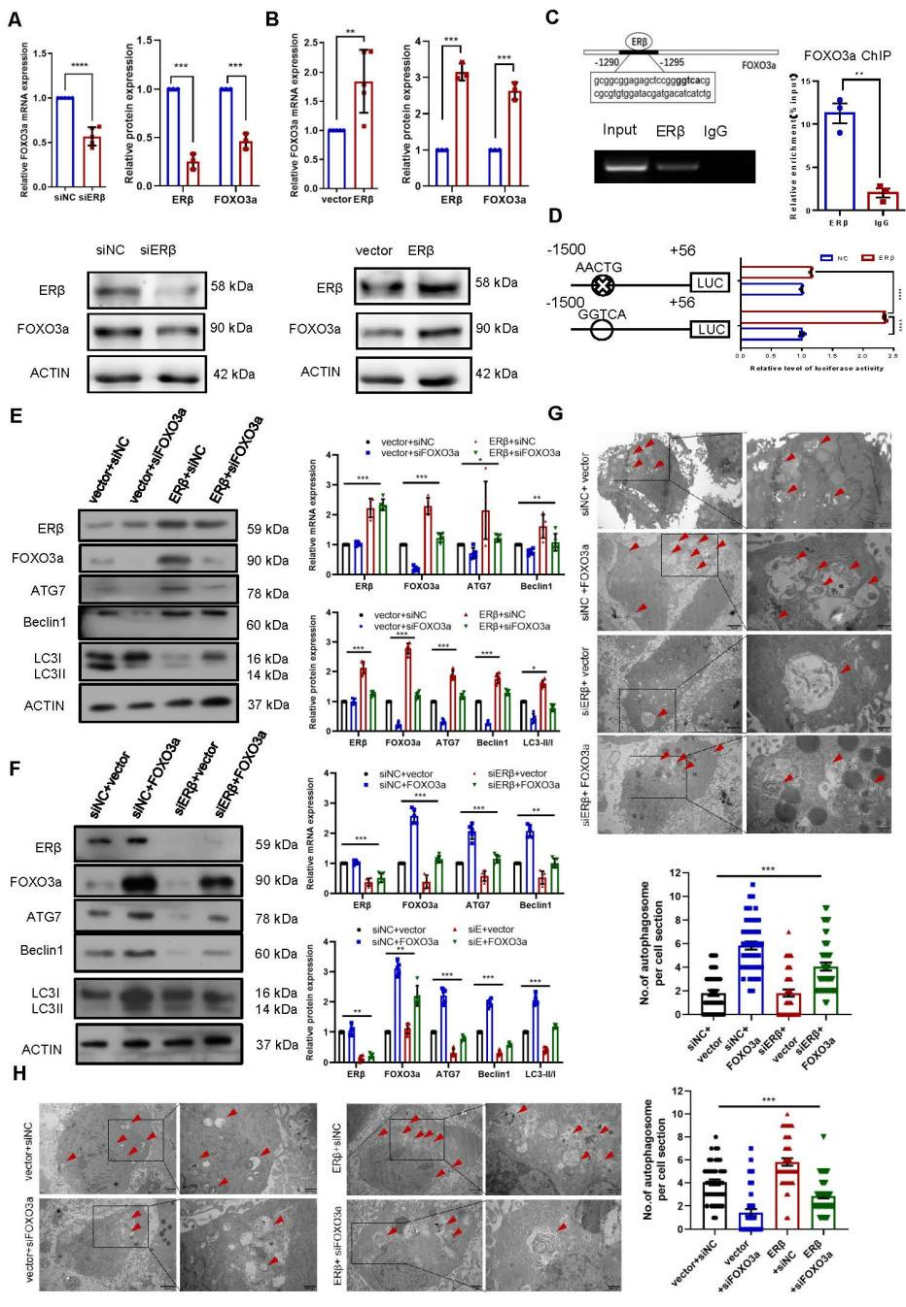
**ERβ-induced autophagy is mediated by FOXO3a**. (**A**) KGN cells were transfected with ERβ siRNA or control siRNA, and FOXO3a expression was measured via qPCR (n=5) or Western blotting (n=3). (**B**) KGN cells were transfected with an ERβ overexpression plasmid or vector, and FOXO3a expression was measured via qPCR (n=5) or Western blotting (n=3). (C KGN cells were harvested and subjected to ChIP using an anti-ERβ or control IgG antibody, followed by SYBR Green-based qPCR (n = 3). (**D**) Reporter plasmids containing the FOXO3a promoter with either a wild-type sequence or a mutated sequence were transiently transfected into KGN cells, after which luciferase activity was measured. The sequences of the indicated mutants were identical to the wild-type sequence except as shown (n = 3). (E, F) KGN cells were transfected with the indicated expression constructs and/or siRNAs, and autophagy-related gene expression was measured by qPCR and Western blotting. The efficiency of the knockdown and overexpression was verified by Western blotting or qPCR. (G, H) KGN cells were transfected with the indicated expression constructs and/or siRNAs, and morphological examination was performed via transmission electron microscopy. The number of autophagosomes was counted in 15 randomly selected cells from each group (n=3). The data are presented as the mean ± SEM. Statistical analyses were carried out by Mann-Whitney U test or two-tailed Student’s t test (*P < 0.05; **P < 0.01; ***P < 0.001; ****P < 0.0001).

### ERβ-induced autophagy is mediated by FOXO3a

Studies have shown that both ERβ and FOXO3a are involved in POF development33, and FOXO3a has been verified to be associated with cell autophagy34; thus, we investigated whether FOXO3a participates in the regulation of autophagy induced by ERβ in GCs. Previous studies have suggested that ERβ can upregulate FOXO3a expression in prostate cancer30. Consistent with these findings, the expression of FOXO3a was decreased (Fig. 3A) by ERβ knockdown but increased (Fig. 3B) by ERβ overexpression. As a transcription factor, ERβ directly regulates the transcription of target genes by binding to estrogen response elements (EREs) in promoter regions. To achieve this goal, we utilized the UCSC (http://genome.ucsc.edu/) and JASPAR (http://jaspardev.genereg.net/) databases to determine the FOXO3a promoter sequence. In this study, we could not find any typical EREs with the sequence 5′-GGTCAnnnTGACC-3′ 35 in the FOXO3a promoter. However, we did discover a potential site for ERβ binding (half-ERE) at -1295/-1290 (Fig. 3C). We conducted a ChIP assay to investigate whether ERβ can bind to the FOXO3a promoter region in KGN cells. ERβ bound to the FOXO3a promoter (Fig. 3C). To validate that ERβ regulates FOXO3a promoter activity, we carried out dual luciferase reporter assays. The results revealed that the promoter activity of the variant with a mutation within the -1295/-1290 region (GGTCA to AACTG) was significantly reduced by 50% compared to that of the wild-type construct (Fig. 3D). Thus, these results indicate that the transcriptional regulatory element ERE, located at -1295/-1290 in the FOXO3a promoter, plays a crucial role in controlling FOXO3a expression in KGN cells.

To further determine whether ERβ affects autophagy via FOXO3a, we first found that FOXO3a knockdown (Fig. 3E and H) reduced autophagy-related gene expression and autophagosome formation, while overexpression (Fig. 3F and G) had the opposite effect. Then we performed rescue experiments and found that ERβ knockdown reduced ATG7, Beclin1, and LC3 expression, and autophagosomes were effectively eliminated by simultaneous overexpression of FOXO3a (Fig. 3F, G). In contrast, the upregulation of the above genes and the increase in autophagosomes caused by ERβ overexpression could be rescued by depletion of FOXO3a (Fig. 3E, H). These observations support the notion that ERβ affects GC autophagy through FOXO3a.

**Figure 4.**
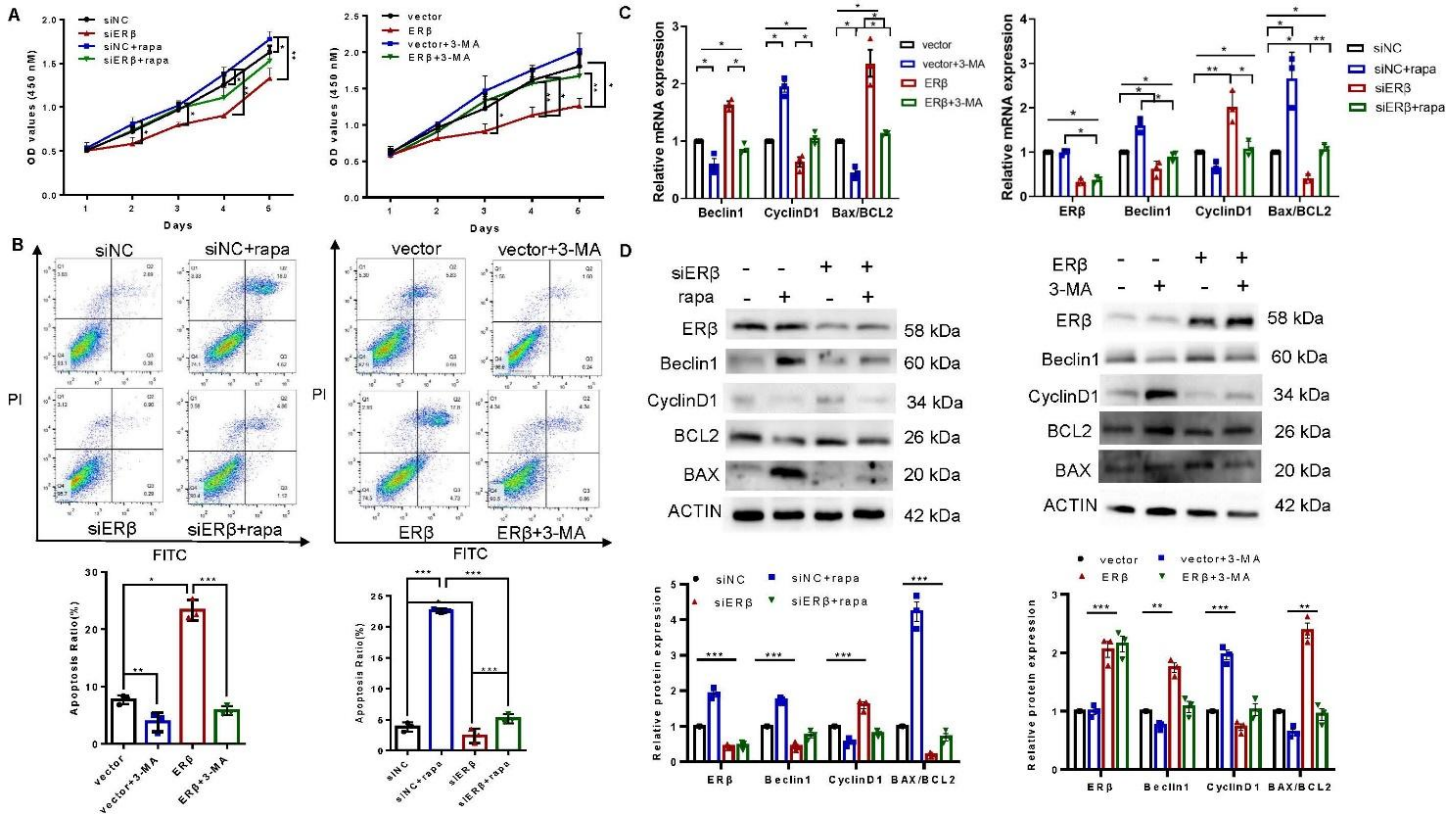
**ERβ promoted cell apoptosis and inhibited cell proliferation by inducing autophagy**. After transfection with the indicated siRNA or plasmid for 24 h, KGN cells were treated with 3-MA or rapa for another 24 h. (**A**) CCK-8 assays were performed to measure KGN cell proliferation (n = 3). (**B**) Flow cytometry analysis was performed to determine the effect of ERβ and autophagy on KGN cell apoptosis. The rate of apoptosis was determined (n=3). (C, D) The mRNA and protein expression levels of the indicated proliferation- and apoptosis-related markers were assessed using qPCR and Western blotting (n=3). The data are presented as the mean ± SEM. Statistical analyses were carried out Mann-Whitney U test. (*P < 0.05; **P < 0.01; ***P < 0.001).

### ERβ promotes cell apoptosis and inhibits cell proliferation by inducing autophagy

We next investigated how ERβ affects GC function using loss- and gain-of-function experiments. Silencing ERβ significantly promoted the proliferation of KGN cells, while ERβ overexpression had the opposite effect, as shown by the CCK-8 assay (Fig. 4A). Moreover, flow cytometry showed that ERβ could facilitate apoptosis, and ERβ deficiency led to a significant decrease in the percentage of apoptotic cells among GCs (Fig. 4B). Furthermore, we assessed the levels of apoptosis and proliferation markers. Silencing ERβ led to an increase in the ratio of BCL2/BAX and in the expression of cyclin D1 (Fig. 4C and D). Upon forced expression of ERβ in KGN cells, the ratio of BCL2/BAX and the expression of cyclin D1 decreased (Fig. 4C and D). To explore the relationship between ERβ-mediated autophagy and apoptosis/ proliferation, we used rapa and 3-MA. Rapa treatment significantly attenuated the increase in proliferation (Fig. 4A) and decrease in apoptosis (Fig. 4B) and markedly reversed the changes in the BCL2/BAX ratio and the expression of cyclin D1 (Fig. 4C and D) induced by ERβ knockdown. In contrast, 3-MA abolished the abovementioned alterations induced by ectopic expression of ERβ (Fig. 4A, B, C, and D). Overall, these results suggest that ERβ triggers cell apoptosis and inhibits cell proliferation in an autophagy-dependent manner.

**Figure 5.**
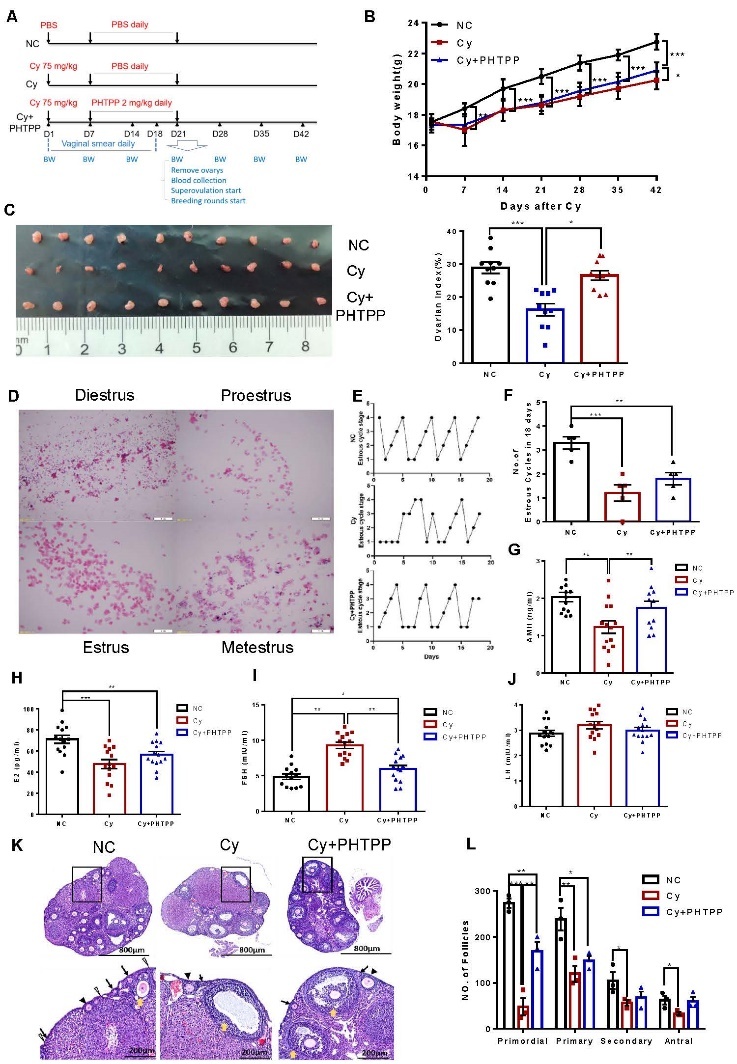
**PHTPP rescued ovarian damage induced by Cy**. (**A**) Design of the experiment in which Cy-induced DOR model mice were treated with PHTPP. B Changes in body weight in each group on days 1, 7, 14, 21, 28, 35 and 42 (n=12). (**C**) Comparison of ovarian morphology and the ovarian indices (ovarian weight [mg]/body weight [g]×100%) (n=10). (**D**) Morphology of typical cells in vaginal smears from mice during different phases of the oestrous cycle following H&E staining; scale bar: 1 mm. (**E**) The oestrous cycle of the mice was observed continuously for 18 days. A representative oestrous cycle from one mouse from each group is shown. The numbers on the x-axis indicate the following: 1, diestrus; 2, proestrus; 3, oestrus; and 4, metestrus. (F During the 18-day observation period, the number of complete oestrous cycles of mice in each group was quantified (n=5). (**G-J**) The levels of AMH, E2, FSH and LH in the serum collected from the three groups were measured via ELISAs (n = 13-14 mice per group). (**K**) Representative images of H&E-stained ovaries from the different groups. Scale bars: 500 μm, 200 μm; white triangle: primary follicle; black arrow: primary follicle; black triangle: secondary follicle; yellow arrow: antral follicle. (**L**) The number of follicles in the different stages in ovary sections from the indicated groups was counted (n = 3 for each group). The data are presented as the mean ± SEM. Statistical analyses were carried out Tamhane T2 test or Kruskal-Wallis H test. (*P < 0.05; **P < 0.01; ***P < 0.001).

### PHTPP rescued ovarian damage induced by Cy

To verify whether the ERβ antagonist PHTPP has a therapeutic effect on DOR in vivo, we established a DOR mouse model in female mice (6-8 weeks old, C57BL/6J) by administering a single intraperitoneal dose of Cy (75 mg/kg, 200-300 μL) for 7 days. As shown in Supplementary Figure 1A, the Cy group had a lower ovarian index and follicle count than did the NC group among female mice with DOR according to histological and morphometric analyses Supplementary (Fig. 1B). After confirming the successful establishment of the DOR model, we determined the protein and mRNA expression of ERβ in ovarian tissues by Western blotting and qPCR analysis (Supplementary Fig. 1C and D). The protein expression of ERβ was upregulated in ovarian tissues from mice with DOR. Consistent with the Western blot results, the qPCR results confirmed that Cy exposure increased the mRNA expression of ERβ. Therefore, ERβ levels were elevated in the ovarian tissues of mice with DOR, which was consistent with what was observed in the GCs of patients with DOR.

**Figure 6.**
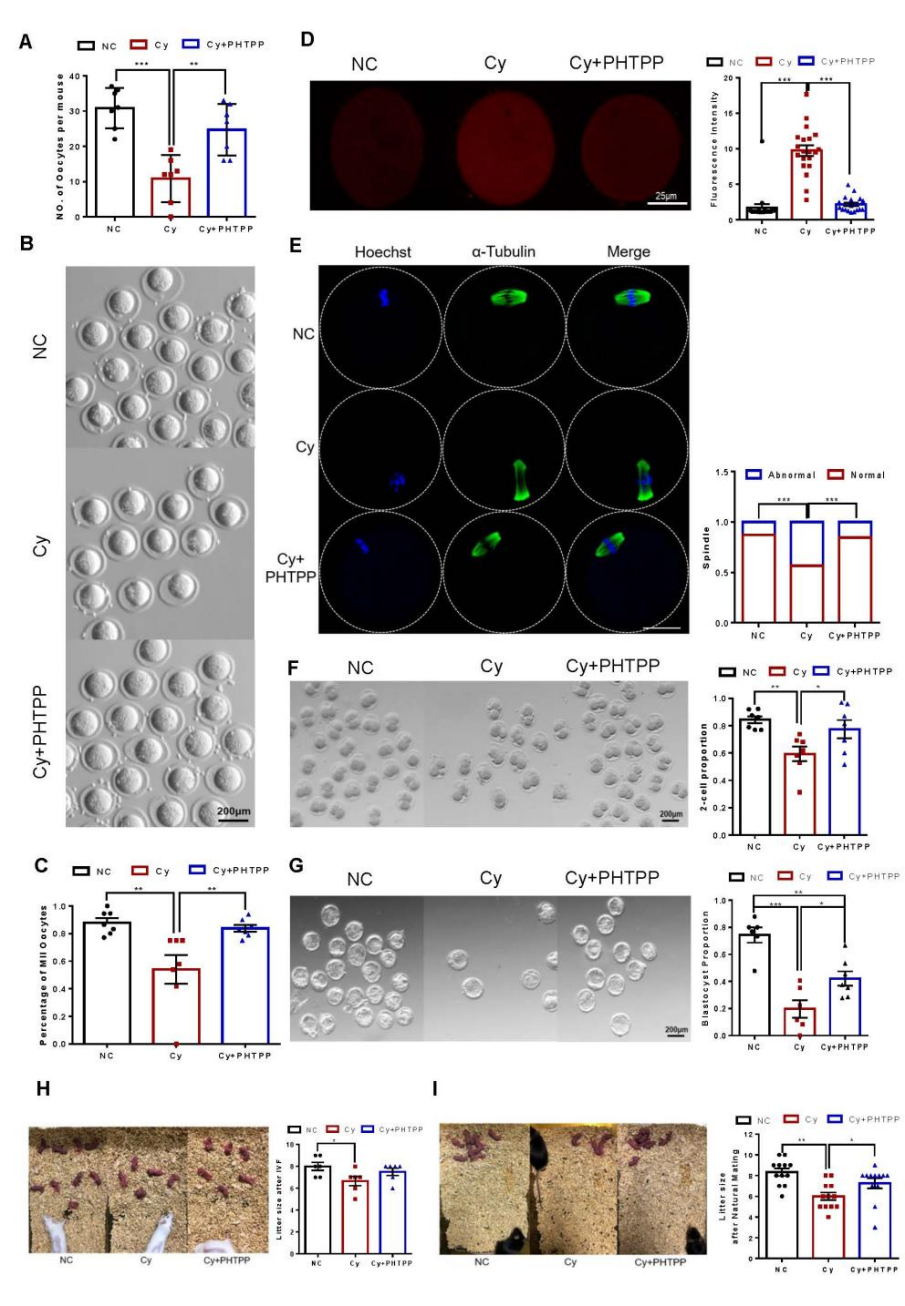
**PHTPP reversed infertility in the Cy-induced DOR model**. (**A**) Quantiffication of ovulated oocytes per mouse in each treatment group (n = 7 mice per group). (**B**) Morphology of MII oocytes from mice in each group; scale bars: 50 μm. C Quantitative analysis of MII oocytes in each treatment group (n=7). (**D**) Measurement of intracellular ROS levels in MII oocytes using MitoSOX staining (red fluorescence); the relative fluorescence intensity was measured (n=20). Scale bars: 25 μm. (**E**) Confocal microscopy results showing the assembly of spindles collected from cultured MII oocytes in each group. Scale bars: 25 μm. The proportion of abnormal MII oocytes with a deformed spindle and misaligned chromosomes was quantified (n=59-73). (**F**) Representative images of 2-cell embryo morphology and statistical analysis of the 2-cell embryo formation rate in the different groups after IVF (n=7). Scale bars: 50 μm. (**G**) Schematic diagram of blastocyst morphology and comparison of blastocyst formation rates among groups (n=6-7). Scale bars: 50 μm. (**H**) Quantiffication of the mean litter size after IVF-ET (n=6). (**I**) Quantiffication of the mean litter size after natural mating (n=12). Statistical analyses were carried out by Tamhane T2 test. (*P < 0.05; **P < 0.01; ***P < 0.001).

After 7 days of DOR induction, the mice were subjected to daily intraperitoneal (i.p.) treatment with PHTPP or vehicle for 14 days (2 mg/kg or 4 mg/kg). Our preliminary experiment revealed that both doses could restored the ovarian reserve (Supplementary Fig. 1E, F and G). Then, based on the results and the principle of minimizing the dosage of drugs, we evaluated the blood physiology and biochemical indices of the mice after intraperitoneal injection at 2mg/kg PHTPP, and the results showed that, compared with those in the NC group, PHTPP itself had little toxicity to the animals and that there were no obvious abnormalities (Supplementary Fig. 1H and I). Thus, we selected 2 mg/kg PHTPP for treatment (the group that received this treatment was referred to as the Cy+PHTPP group).

Animal experiments were performed according to the experimental timeline presented in Figure 5A. After PHTPP treatment, mice in the Cy+PHTPP group exhibited gradual weight gain and significant increases in both ovarian weight and the ovarian indices, as shown in Fiure. 5B and C. We subsequently monitored the oestrous cycle, and the results showed that PHTPP partially restored the estrous cycle in mice with DOR (Fig. 5D and F). Treatment with PHTPP reversed the abnormalities in blood AMH and FSH levels but had no effect on E2 or LH levels (Fig. 5G, H, I and J). Moreover, ovarian morphology was compared among the NC, Cy, and Cy+ PHTPP groups, and H&E staining (midline sections) of ovaries showed that PHTPP treatment signifficantly alleviated Cy-induced damage to ovarian follicles, contributing to their normal development (Fig. 5K and L). Quantitative analysis revealed that treatment with PHTPP reduced the Cy-induced decrease in primordial and primary follicle counts (Fig. 5L). Taken together, these results indicate that PHTPP can rescue ovarian damage induced by Cy.

### PHTPP reverses infertility in the Cy-induced DOR model

In mammals, the development of both oocytes and follicles regulates the reproductive capacity of females36. The number and quality of oocytes are crucial for fertility. Administering PHTPP increased the number of ovulated oocytes, as shown in Fig. 6A. For evaluation of the developmental potential of the oocytes, germinal vesicle (GV) oocytes from the NC, Cy, and Cy+PHTPP groups were isolated and cultured in vitro. The MII rate of Cy oocytes was significantly decreased, while there were no differences in the MII rate between Cy+PHTPP and NC group oocytes (Fig. 6B and C). Furthermore, we examined the level of ROS in the NC, Cy and Cy+PHTPP groups of oocytes. Cy mice exhibited signifficantly increased ROS production, and a dramatic decrease in ROS levels was observed in oocytes from the Cy+ PHTPP group (Fig. 6D). In addition, abnormal spindle assembly, loss of normal morphology, loss of polarity and disordered chromosome arrangement were observed in oocytes from the Cy-treated mice (Figure 6E). However, the percentage of MII oocytes exhibiting abnormal spindles was significantly lower in the mice treated with Cy+PHTPP (Fig. 6E). These results indicate that the increase in ERβ levels not only impairs follicular development but also alters oocyte development.

To explore the developmental potential of oocytes in the different groups, we conducted IVF using a previously described method37. The results showed that the rate of 2-cell embryo formation was notably lower in the Cy group than in the NC group. However, the level was significantly greater than that in the Cy+PHTPP group (Fig. 6F). Then, 2-cell embryos were cultured in vitro, after which the blastocyst formation rate was calculated. The results showed that the blastocyst formation rate of the mice in the Cy group was also significantly lower than that of the mice in the NC group and was significantly greater after PHTPP treatment (Fig. 6G). IVF-ET and mating trials were subsequently performed to further determine the protective effect of PHTPP on fertility. We observed that the litter size (number of pups per litter) in the Cy group was notably lower than that in the NC group after IVF-ET (Fig. 6H), while the litter size in the PHTPP-treated group was greater than that in the Cy group; however, the difference was not significant (Fig. 6H). Furthermore, compared with those of the NC group, the litters of the Cy group exhibited a reduced size after natural mating. Importantly, this change in litter size was reversed by PHTPP treatment (Fig. 6I). These results suggest that the Cy-induced fertility defects can be effectively rescued by PHTPP treatment.

### Effects of PHTPP on autophagy and apoptosis in the ovaries of DOR model mice

**Figure 7.**
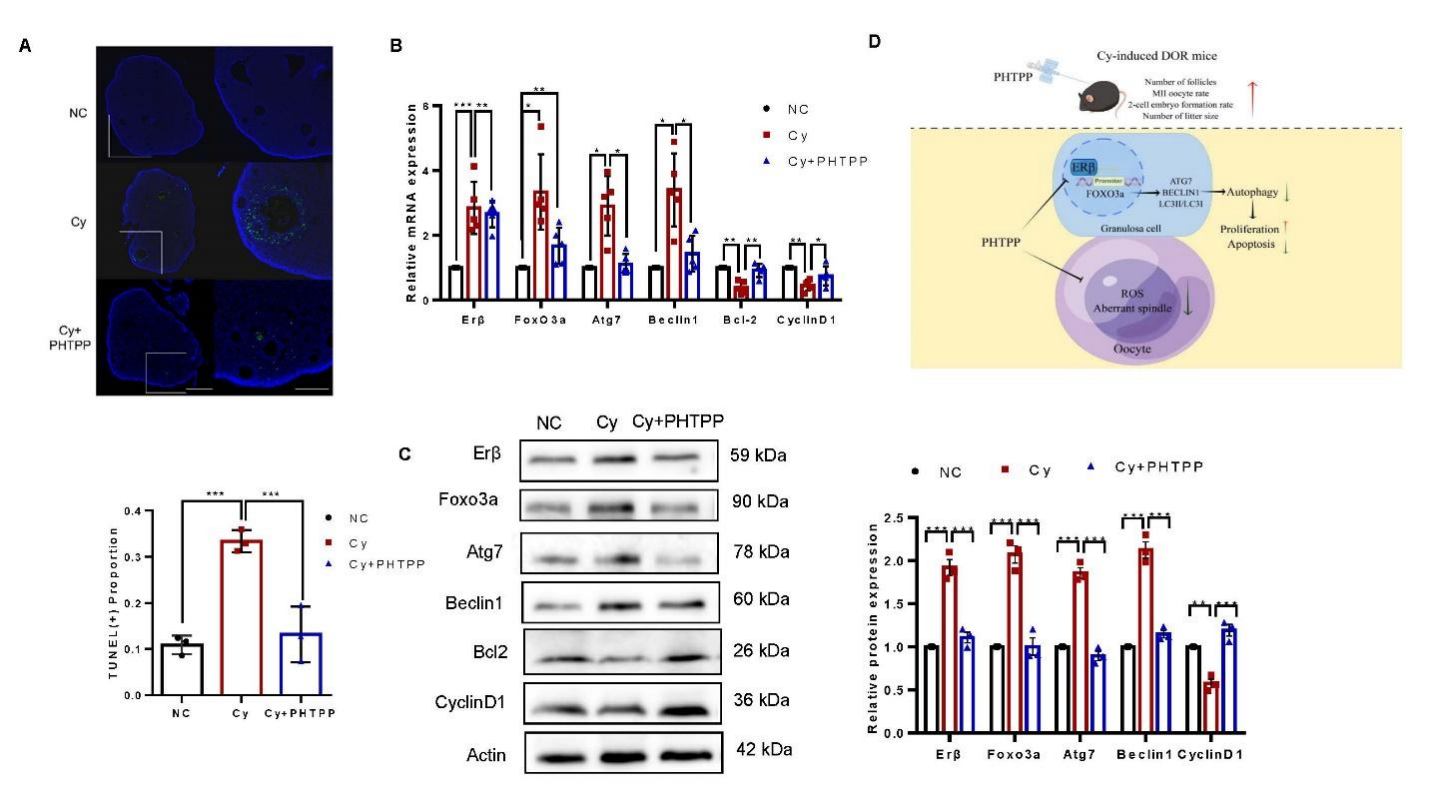
**Effects of PHTPP on apoptosis and autophagy in the ovaries of DOR model mice**. (**A**) TUNEL staining was performed to evaluate GC apoptosis in the ovaries of each treatment group, and TUNEL-positive cells in the ovarian sections were quantitatively analysed (n = 3 for each group). Scale bars: 500 µm, 200 µm. (B, C) Ovaries were obtained from mice in each treatment group. The mRNA and protein expression levels of Erβ, Foxo3a, Atg7, Beclin1, Bcl-2 and Cyclin D1 were measured by qPCR (n=5) and Western blotting (n=5). (**D**) Schematic showing the role of ERβ in the regulation of FOXO3a/autophagy signalling in GCs. Statistical analyses were carried out by Kruskal-Wallis H test. (*P < 0.05; **P < 0.01; ***P < 0.001).

In the Cy group, many follicles exhibited significant GC apoptosis. However, these changes were not as severe in the ovaries of mice that received PHTPP treatment, as illustrated in Figure 7A. We quantified the labelled cells after TUNEL staining. The results indicate that, compared with the control treatment, Cy treatment increased the number of TUNEL-positive follicles, which is an indicator of an increased apoptotic index. PHTPP protected against this increase in the apoptotic index, as the apoptotic index of follicles from the Cy + PHTPP group was significantly lower than that of follicles from the Cy group and similar to that of follicles from the control group.

We analysed the expression of genes related to the FOXO3a/autophagy pathway, apoptosis, and proliferation in mouse ovaries following Cy treatment with or without PHTPP to examine follicle activation mechanisms. Cy treatment resulted in an increase in FOXO3a, ATG7 and Beclin1 expression, but PHTPP inhibited this increase (Fig. 7B). Furthermore, PHTPP treatment increased the expression of Bcl-2 (Fig. 7B), suggesting that PHTPP inhibited the decrease in follicle count caused by Cy-induced apoptosis. Similar effects on the protein levels of the above genes were also observed (Fig. 7C). These results further suggest that PHTPP can reduce autophagy and apoptosis and promote proliferation in the ovaries of mice with DOR. The present study suggested that the therapeutic effects of PHTPP in humans are comparable to those in mice.

## DISCUSSION

In recent years, the occurrence of DOR has been increasing, and DOR tends to affect younger patients1. DOR is a popular research topic in the field of reproductive health, and different techniques, including mitochondrial transplantation, primordial follicle activation, in vitro follicle culture and stem cell transplantation, are being explored to improve the quality and quantity of oocytes in patients with DOR38-40; however, there are still no ideal treatment options available for women who suffer from DOR. In this study, we found for the first time that ERβ/FOXO3a/autophagy plays an important role in the etiopathogenesis of DOR. Moreover, a selective ERβ antagonist, PHTPP, was found to partially improve ovarian function and pregnancy outcomes in Cy-induced DOR model mice. Taken together, these results demonstrate that PHTPP has beneficial effects on a mouse model of DOR and provide an experimental basis for further exploration of its therapeutic potential in patients with DOR.

DOR refers to a decrease in the number and/or quality of oocytes. The number of oocytes is affected by the size of the primordial follicle pool, follicle growth and development, maturation, ovulation and follicle atresia1. Follicular atresia is a crucial selective process in the development of mammalian ovarian follicles and is the main cause of oocyte loss, which occurs in association with GC death16. Autophagy is a highly regulated intracellular process that helps maintain cellular homeostasis, protects against starvation, aids in differentiation, and supports normal growth41. Increasing evidence indicates that autophagy in GCs is involved in follicular atresia42. Choi et al. reported that autophagy is induced mainly in GCs during follicular atresia, leading to apoptotic cell death15, 43. These findings imply that autophagy plays a direct role in follicular atresia and has a negative effect on the ovarian reserve. Over the years, multiple reports have indicated that autophagy plays a role in regulating oocyte survival and the formation of primordial follicles. However, conflicting results have been reported by different groups, and the findings seem to be dependent on various factors, such as the type of mouse strain used, the method of inducing or inhibiting autophagy, the concentration of the agent used, and the duration of exposure44. Therefore, assessing the difference in autophagy levels between GCs from patients with DOR and those from patients with normal ovarian reserve function is crucial for revealing the effect of autophagy on the ovarian reserve. We found that the expression of the autophagy-related factors ATG7 and Beclin1 and the LC3II/LC3I ratio were increased in the GCs of patients with DOR, suggesting that autophagy was overactivated. The above results were further confirmed by electron microscopy. However, the molecular pathways controlling the induction of autophagy in GCs are unclear.

In the ovary, GCs are responsible for estrogen synthesis45. Research has demonstrated that ERβ is the major oestrogen receptor isoform in the adult female ovary, particularly in GCs24, 25; therefore, it is highly probable that ERβ plays an important role in the regulation of ovarian function. At present, findings related to the effect of ERβ on the ovarian reserve are inconsistent. On the one hand, ERβ may play a crucial role in maintaining the primordial follicle pool. A study on the effect of gamma radiation on the ovarian reserve in rats revealed that upregulation of ERβ expression in the ovary could inhibit the transition of primordial follicles to more growing follicles and promote GC proliferation46. On the other hand, ERβ inhibits GC and oocyte development. Nerve growth factor affects intracellular signalling cascades and cyclin integration by suppressing ERβ expression, thereby inhibiting GC proliferation and affecting follicle quality47. Another study revealed that activation of ERβ during mid-pregnancy in mice results in delayed primordial follicle development after birth, impaired follicle development after prepuberty, and ultimately a reduced total litter size48. Furthermore, studies involving Esr2-/- rats have suggested that ERβ plays a gatekeeping role in maintaining primordial follicle activation49. In our current research, we also discovered that ERβ may have a detrimental effect on ovarian reserve. We observed a significant increase in ERβ expression in both patients with DOR and Cy-induced DOR model mice, but the specific regulatory mechanism involved is still unclear.

The autophagy-inducing function of ERβ has been confirmed in various cell types, such as osteosarcoma cells and human seminoma cells26, 29. In breast cancer cells, ERβ can increase autophagic flux by reducing the expression of BCL-2 and promoting cancer cell death50. However, the relationship between ERβ and autophagy in human or mouse GCs or ovaries has not been reported. Here, we found that ERβ can activate autophagy in GCs, promote cell apoptosis and inhibit cell proliferation through autophagy, suggesting that the decrease in the ovarian reserve caused by high ERβ expression is achieved by the promotion of autophagic death in GCs.

Subsequently, we investigated the molecular mechanisms of ERβ-regulated autophagy in GCs. In breast cancer cells and prostate cancer cell lines, ERβ can upregulate FOXO3a expression, but the mechanism is not yet clear30, 51. FOXO3a is an important intraovarian signalling molecule that negatively regulates oocyte growth and follicular development. Research has shown that the FOXO3a protein contributes to follicular atresia in mammalian ovarian GCs and oocytes by promoting apoptosis22, 52, suggesting that FOXO3a might be a candidate gene for the development of DOR. Consistent with our hypothesis, patients with DOR exhibited high FOXO3a expression, and FOXO3a expression was positively correlated with ERβ expression. Given these ffindings, we further demonstrated that ERβ can play a role in transcriptionally activating FOXO3a and that the ERE sequence located at -1290/-1295 in the FOXO3a promoter is crucial for regulating FOXO3a expression. Additionally, FOXO3a is a potent transcriptional activator that induces the expression of autophagy-related genes. FOXO3a can regulate autophagy in skeletal muscle cells by transcriptionally activating autophagosome formation-related genes, including LC3 and Beclin153. Moreover, ATG7, a gene involved in autophagy, is regulated by FOXO3a in non-small cell lung cancer cells54. The above study suggested that FOXO3a regulates autophagy by controlling the expression of autophagy-related genes. However, whether FOXO3a affects ovarian and GC quality by changing the level of autophagy and subsequently affecting ovarian reserve function has not been studied. In this study, we discovered that ERβ targets FOXO3a to stimulate autophagy in ovarian GCs.

Then, the ERβ-specific antagonist PHTPP was administered, and its therapeutic effect on mice with DOR was evaluated in vivo. PHTPP has been proven to effectively treat breast cancer and endometriosis both in laboratory and animal studies55, 56. We investigated the therapeutic effect of this herb on DOR for the first time. The DOR animal model is relatively mature and variable and includes mainly chemical drugs, radiation, genetic knockout, and the induction of ovarian immune inflammation 57-60. Considering the reproducibility, time consumption, success rate and other factors, the Cy modelling method for chemical drugs is relatively mature, reliable, simple, and feasible, has a short construction period, can provide a window for subsequent administration of therapeutic drugs, and is widely used in basic research61-63. Therefore, in the present study, intraperitoneal injection of Cy was chosen to establish a DOR model. Our results, as well as those of previous studies64, indicate that treatment with 75 mg/kg Cy induces DOR-like damage to the ovary. The ovarian index is the ratio of the ovarian wet weight to the body weight and is one of the indices used to evaluate ovarian function65. This study revealed that PHTPP can increase the ovarian index, suggesting that PHTPP can ameliorate ovarian atrophy caused by Cy and restore ovarian function to a certain extent. Cy has been reported to affect the secretion of steroid hormones by increasing apoptosis and altering follicular development, leading to ovarian dysfunction66, which manifests as low levels of E2 and high levels of FSH67. The results we observed in this study were consistent with those of the above studies, as PHTPP reduced the serum FSH level and increased the serum E2 level in mice with DOR, indicating that PHTPP helps to improve and restore ovarian endocrine function. AMH is a reliable marker of ovarian reserve and can indicate follicle pool size68. We discovered that the decrease in primordial and primary follicles in the Cy group correlated with lower levels of serum AMH. After the administration of PHTPP, the number of primordial follicular pools was restored, and the AMH level was increased, suggesting that PHTPP restored the ovarian reserve to some extent.

Both reduced oocyte quantity and quality can lead to DOR. Cytotoxicity of Cy can lead to apoptosis of follicles at all levels. Although oocyte apoptosis induced by DNA damage resulting from Cy generally occurs within 2 days, GC damage has a superimposed effect on follicle development, leading to premature atresia of follicles and prolonged duration of apoptosis. This phenomenon is also the reason why ovarian GC apoptotic signals can be detected 2-3 weeks after the administration of Cy64, 69, 70. In the present study, more apoptotic follicles containing many TUNEL-positive GCs were observed in the 75 mg/kg Cy group than in the control group, and the level of Bcl-2, which suppresses apoptosis, was also reduced in the ovaries. After PHTPP treatment, apoptosis was decreased in mice with DOR, suggesting that in addition to ERβ expression, ovarian apoptosis was also inhibited in mice with DOR, thus alleviating the adverse effects on follicular development. Furthermore, there are many factors that cause oocyte quality to decrease, including increased oxidative stress, faulty spindle assembly, mitochondrial dysfunction, gene mutations, meiotic abnormalities, and decreased chromosome cohesion71. All of these factors can adversely affect oocyte development individually or collectively. In the present study, the administration of the ERβ inhibitor PHTPP reduced the ROS level in oocytes, reduced the damage caused by oxidative stress in oocytes, and promoted the recovery of oocyte quality. This finding is consistent with the increase in ROS levels induced by E2 through ERβ in human seminoma cell lines72. The proportion of MII eggs with chromosome arrangement and abnormal spindle morphology was greater in older women (40-45 years) than in younger women (20-25 years) (79% vs. 27%)73. This finding suggested that spindle assembly may be abnormal when oocytes age and when their quality decreases. In this study, we found that the inhibition of high ERβ expression may be related to the recovery of spindle formation, suggesting that PHTPP can also promote the improvement of MII egg quality by improving the spindle assembly process.

Both an increase in ROS levels and abnormal spindle assembly can cause a decrease in oocyte quality and a decrease in developmental potential. Therefore, under the premise that sperm quality was normal, and the fertilization environment was consistent between the different groups, the 2-cell embryo and blastocyst formation rates of the Cy group decreased, suggesting the poor potential of oocytes to develop into embryos and the decreased quality of embryos in the Cy group. After the application of PHTPP, the proportion of 2-cell embryos and blastocysts increased. These findings suggested that PHTPP can improve the quality of oocytes, restore their developmental potential, and improve embryonic development in mice with DOR. Moreover, the litter size of mice with DOR increased following natural mating, suggesting that the fertility of mice with DOR also improved after PHTPP administration.

There are still many limitations in this study. Although many studies have confirmed its feasibility, the toxicity of chemical drugs itself does not rule out an effect on oocytes and granulosa cells in the ovary, which may not be able to truly simulate DOR in the population not receiving chemotherapy. In addition, in the present study, we investigated the effect of PHTPP treatment on the quality of granulosa cells and oocytes, but whether the effect on oocytes is caused by direct or indirect effects through gap junctions remains to be further explored. ERβ was the core factor identified in this study, and how its isoforms play a role in DOR is also worth exploring.

Here, we elucidated the molecular mechanism by which ERβ induces ovarian GC apoptosis through the FOXO3a/autophagy pathway and confirmed the protective effect of the ERβ antagonist PHTPP on ovarian reserve and fertility in vivo. These results suggest that PHTPP may be a promising medication for treating DOR in a clinical setting. In the future, an ovary-specific ERβ gene knockout mouse model needs to be constructed to obtain more direct and reliable evidence.

## Supplementary Materials

The Supplementary data can be found online at: www.aginganddisease.org/EN/10.14336/AD.2024.0221.


