# Supplementary material for: Targeting Estrogen Receptor Beta Ameliorates Diminished Ovarian Reserve via Suppression of the FOXO3a/Autophagy Pathway
Source: Aging Dis. 2024 Feb 21;16(1):479–97. doi: 10.14336/AD.2024.0221 (PMC11745447; doi:10.14336/AD.2024.0221)
Supplement: Supplementary file 1 [file AD-16-1-479-s.pdf]

SUPPLEMENTARY DATA

Targeting Estrogen Receptor Beta Ameliorates Diminished Ovarian Reserve via Suppression of the FOXO3a/Autophagy Pathway

Fangyuan Li, Jingwen Zhu, Jinchen Liu, Yongyan Hu, Peili Wu, Cheng Zeng, Ruihui Lu, Ning Wu, Qing Xue

© 2024. Li F et al. Published online at http://www.aginganddisease.org/EN/10.14336/AD.2024.0221

SUPPLEMENTARY DATA

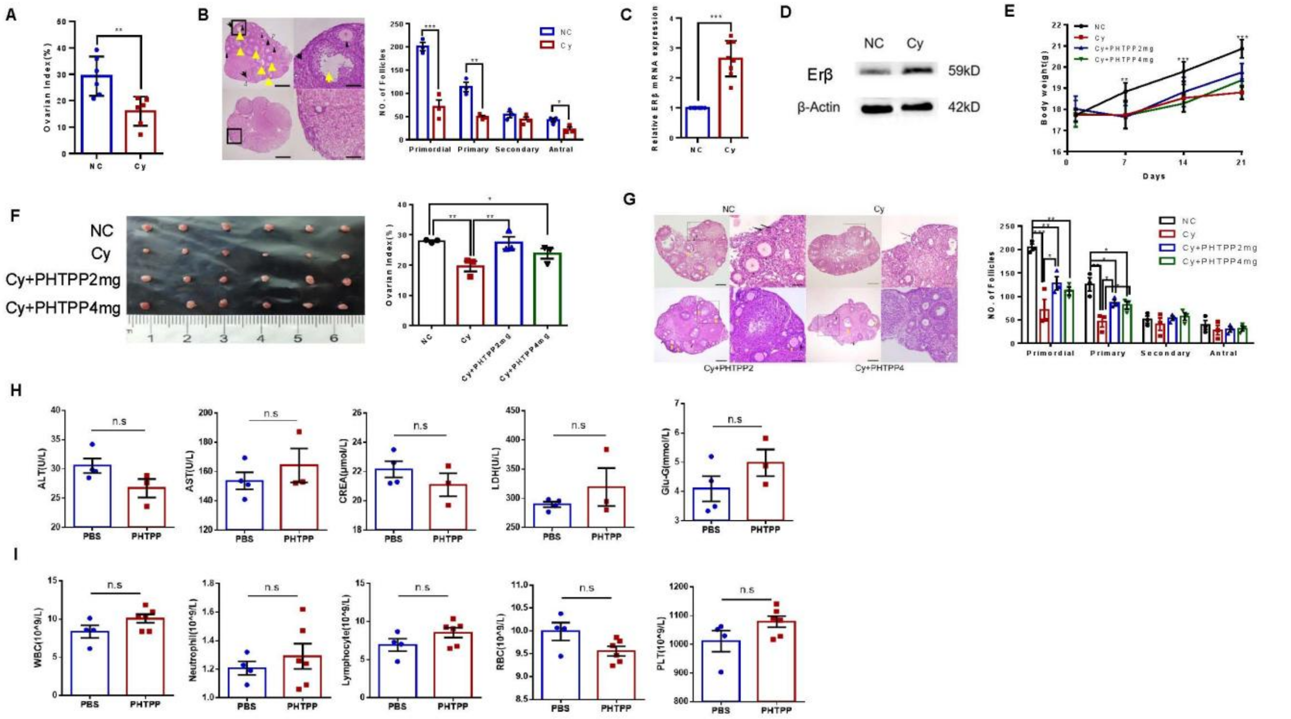

Supplementary Figure 1. PHTPP rescued ovarian damage induced by Cy. A Comparison of ovarian indices (ovarian weight [mg]/body weight [g]×100%) (n=6). **B** Representative images of H&E-stained ovaries from the two groups. Scale bars: 500 μm, 200 μm. The number of follicles in the different stages in ovary sections from the indicated groups was counted (n = 3 for each group). **C, D** Ovaries were obtained from mice in each treatment group. The mRNA and protein expression levels of Erβ were measured by qPCR (n=8) and Western blotting (n=5), respectively. **E** Changes in body weight in each group on days 1, 7, 14, and 21 (n=6). **F** Comparison of ovarian morphology and the ovarian indices (ovarian weight [mg]/body weight [g]×100%) (n=6). **G** Representative images of H&E-stained ovaries from the two groups. Scale bars: 500 μm, 200 μm. The number of follicles in the different stages in ovary sections from the indicated groups was counted (n = 3 for each group). **H** The blood level of ALT, AST, CREA, LDH and Glu in the mice after treatment with PHTPP. **I** blood routine analysis in the mice after treatment with PHTPP. The data are presented as the mean ± SEM. Statistical analyses were carried out by Kruskal-Wallis H test or Mann-Whitney U test. (*P < 0.05; **P < 0.01; ***P < 0.001).

© 2024. Li F et al. Published online at http://www.aginganddisease.org/EN/10.14336/AD.2024.0221
